# Usage and applications of Semantic Web techniques and technologies to support chemistry research

**DOI:** 10.1186/1758-2946-6-18

**Published:** 2014-04-28

**Authors:** Mark I Borkum, Jeremy G Frey

**Affiliations:** 1 Chemistry, Faculty of Natural & Environmental Sciences, University of Southampton, Highfield, Southampton, SO17 1BJ, UK

**Keywords:** Chemical information, Linked data, Resource Description Framework (RDF), Semantic web

## Abstract

**Background:**

The drug discovery process is now highly dependent on the management, curation and integration of large amounts of potentially useful data. Semantics are necessary in order to interpret the information and derive knowledge. Advances in recent years have mitigated concerns that the lack of robust, usable tools has inhibited the adoption of methodologies based on semantics.

**Results:**

This paper presents three examples of how Semantic Web techniques and technologies can be used in order to support chemistry research: a controlled vocabulary for quantities, units and symbols in physical chemistry; a controlled vocabulary for the classification and labelling of chemical substances and mixtures; and, a database of chemical identifiers. This paper also presents a Web-based service that uses the datasets in order to assist with the completion of risk assessment forms, along with a discussion of the legal implications and value-proposition for the use of such a service.

**Conclusions:**

We have introduced the Semantic Web concepts, technologies, and methodologies that can be used to support chemistry research, and have demonstrated the application of those techniques in three areas very relevant to modern chemistry research, generating three new datasets that we offer as exemplars of an extensible portfolio of advanced data integration facilities. We have thereby established the importance of Semantic Web techniques and technologies for meeting Wild’s fourth “grand challenge”.

## Introduction

In the inaugural issue of the Journal of Cheminformatics, Wild identified [1] four “grand challenge” areas for cheminformatics, of which the fourth is particularly pertinent to this article: 

“Enabling the network of the world’s chemical and biological information to be accessible and interpretable.”

The drug discovery process is now highly dependent on the management, curation, and integration of large amounts of potentially useful data. A year before Wild’s publication, Slater et al. argued [2] that it is not sufficient to simply bring together data and information from multiple sources; semantics are necessary in order to interpret the information and derive knowledge. They proposed a knowledge representation scheme that matches the Semantic Web vision of data and resource descriptions readable by both humans and machines [3,4].

At about the same time, Chen et al. published a survey of semantic e-Science applications [5], opening their conclusion with the following statement: 

“As semantic technology has been gaining momentum in various e-science areas, it is important to offer semantic-based methodologies, tools, middleware to facilitate scientific knowledge modeling [sic], logical-based hypothesis checking, semantic data integration and application composition, integrated knowledge discovery and data analyzing [sic] for different e-science applications.”

During the four years since the publication of Wild’s article, it has become increasingly important to adopt an inclusive view. The need to discover and access “the world’s chemical and biological information” now extends far beyond drug discovery. For example, chemical information is ever more germane to the development of new materials, to advances in medicine, and to the understanding of environmental issues, especially those related to atmospheric chemistry.

Advances in recent years have mitigated concerns that the lack of robust, usable tools has inhibited the adoption of methodologies based on semantics. Frey and Bird have recently reviewed [6] the progress made by cheminformatics towards the goals of integration, owing to the influence of Semantic Web technologies.

Losoff, writing from the perspective of a science librarian, reasoned [7] that integrating databases with other resources, including journal literature, was important for furthering scientific progress. She explored the role of semantics and discussed the role for librarians in data curation. Bird and Frey discuss [8] the importance of curation for chemical information, together with the associated concepts of preservation, discovery, access, and provenance.

From the outset in 2000 of the UK e-Science programme [9], the University of Southampton has studied how Semantic Web techniques and technologies can be used to support chemistry research. Building on early, text- and eXtensible Markup Language (XML)-based formats for the exposition of chemical information [10,11], the Frey group has investigated [12-18] the application of Resource Description Framework (RDF) and other Semantic Web technologies to the capture, curation and dissemination of chemical information.

Recent research conducted by the Frey group has benefitted considerably from the development of modern, high quality chemical ontologies [19,20] and the availability of open-access, online chemical databases [21]. Leveraging these information resources, projects such as oreChem [22] have explored the formalisation of laboratory-based protocols and methodologies through the exposition of both prospective and retrospective provenance information (machine-processable descriptions of the researcher’s intentions and actions); an approach that has since been applied [23] to retrospectively enhance “ancient” data from other projects.

Chemists and the cheminformatics community have thus been aware for several years of the requirement for advanced data integration facilities in scientific software systems. Recent years have seen a growing realisation of the importance of semantics and the relevance of Semantic Web technologies. For example, Chepelev and Dumontier have implemented Chemical Entity Semantic Specification (CHESS) for representing chemical entities and their descriptors [24]. A key aim for CHESS is to facilitate the integration of data derived from various sources, thereby enabling more effective use of Semantic Web methodologies.

Advanced data integration requires the ability to unambiguously interpret conceptual entities such that data may be shared and re-used at any time in the future. Given this ability, data never loses its value, and hence, it is always possible to extract new value from old data, by integrating it with new data.

Semantic Web technologies enable data integration by allowing the structure and semantics of conceptual entities to be fixed, e.g., as controlled vocabularies, taxonomies, ontologies, etc. Hence, we argue that it is of vital importance that the cheminformatics community (and the chemistry community in general) endorses the use of Semantic Web techniques and technologies for the representation of scientific data.

In this article, our goal is to demonstrate how Semantic Web techniques and technologies can be used in order to support chemistry research. Accordingly, the remainder of this article is organised as follows: First, we introduce the Semantic Web, along with the vocabularies that we intend to use for our examples. Second, we present four examples of the use of Semantic Web techniques and technologies (three datasets and one software application). Third, we discuss the legal implications of the use of Semantic Web technologies in an environment that is hazardous to health, e.g., a laboratory. This is followed by an evaluation and discussion of our approach. Finally, the article is concluded.

## Background

In this section we introduce the Semantic Web and discuss the associated techniques and technologies for knowledge representation.

### Semantic Web

The Semantic Web is a collaborative movement that argues for the inclusion of machine-processable data in Web documents [3]. The goal of the Semantic Web movement is to convert the information content of unstructured and semi-structured Web documents into a “Web of data” [25] for consumption by both humans and machines. The activities of the Semantic Web movement are coordinated by the World Wide Web Consortium (W3C) [26], and include: the specification of new technologies; and, the exposition of best practice.

The architecture of the Semantic Web, commonly referred to as the “layer cake” [27], is a stack of technologies, where successive levels build on the capabilities and functionality of prior levels.

At the base of the stack is the Uniform Resource Identifier (URI)—a string of characters that is used to identify a Web resource. Such identification enables interaction with representations of the Web resource over a network (typically the World Wide Web) using specific protocols.

At the next level of the stack is the RDF [28,29]—a family of specifications, which collectively define a methodology for the modelling and representation of information resources as structured data.

In RDF, the fundamental unit of information is the subject-predicate-object tuple or “triple”. Each triple encapsulates the assertion of a single proposition or fact, where: the “subject” denotes the source; the “object” denotes the target; and, the “predicate” denotes a verb that relates the source to the target.

In RDF, the fundamental unit of communication (for the exchange of information) is the unordered set of triples or “graph”. According to the RDF semantics [29], any two graphs may be combined to yield a third graph.

Using a combination of URIs and RDF, it is possible to give identity and structure to data. However, using these technologies alone, it is not possible to give semantics to data. Accordingly, the Semantic Web stack includes two further technologies: RDF Schema (RDFS) and the Web Ontology Language (OWL).

RDFS is a self-hosted extension of RDF that defines a vocabulary for the description of basic entity-relationship models [30]. RDFS provides metadata terms to create hierarchies of entity types (referred to as “classes”) and to restrict the domain and range of predicates. However, it does not incorporate any aspects of set theory, and hence, cannot be used to describe certain types of models.

OWL is an extension of RDFS, based on the formalisation of description logics [31], which provides additional metadata terms for the description of arbitrarily complex entity-relationship models, which are referred to as “ontologies”.

### Commonly-used vocabularies

In this section we briefly introduce three popular vocabularies that are used in order to construct our datasets.

#### Dublin core

The Dublin Core Metadata Initiative (DCMI) is a standards body that focuses on the definition of specifications, vocabularies and best practice for the assertion of metadata on the Web. The DCMI has standardised an abstract model for the representation of metadata records [32], which is based on both RDF and RDFS.

The DCMI Metadata Terms is a specification [33] of all metadata terms that are maintained by the DCMI, which incorporates, and builds upon, fifteen legacy metadata terms, defined by the Dublin Core Metadata Element Set, including: “contributor”, “date”, “language”, “title” and “publisher”.

In the literature, when authors use the term “Dublin Core”, they are most likely referring to the more recent DCMI Metadata Terms specification.

Our decision to use DCMI Metadata Terms is motivated by the fact that, today, it is the *de facto* standard for the assertion of metadata on the Web [34]. Accordingly, metadata that is asserted by our software systems using DCMI Metadata Terms can be easily integrated with that of other software systems.

#### OAI-ORE

Resources that are disseminated on the Web do not exist in isolation. Instead, some resources have meaningful relationships to other resources. An example of a meaningful relationship is being “part of” another resource, e.g., a supplementary dataset, figure or table is part of a scientific publication. Another example is being “associated with” another resource, e.g., a review is associated with a scientific publication. When aggregated, these entities and their relationships form a “compound object” that can be consumed and manipulated as a whole, instead of in separate parts, by automated software systems.

The goal of the Open Archives Initiative Object Reuse and Exchange (OAI-ORE) is “to define standards for the description and exchange of aggregations of Web resources” [35]. The OAI-ORE data model addresses two issues: the assertion of identity for both aggregations and their constituents, and the definition of a mechanism for the assertion of metadata for either the aggregation or its constituents.

Our decision to use OAI-ORE is motivated by the fact that, like DCMI Metadata Terms, OAI-ORE is emerging as a *de facto* standard for the implementation of digital repositories [36,37].

#### SKOS

The goal of the Simple Knowledge Organization System (SKOS) project is to enable the publication of controlled vocabularies on the Semantic Web, including, but not limited to, thesauri, taxonomies and classification schemes [38]. As its name suggests, SKOS is an organisation system that relies on informal methods, including the use of natural language.

The SKOS data model is based on RDF, RDFS and OWL, and defines three main conceptual entities: concept, concept scheme and collection. A concept is defined as a description of a single “unit of thought”; a concept scheme is defined as an aggregation of one or more SKOS concepts; and, a collection is defined as a labelled and/or ordered group of SKOS concepts.

In SKOS, two types of semantic relationship link concepts: hierarchical and associative. A hierarchical link between two concepts indicates that the domain is more general (“broader”) than the codomain (“narrower”). An associative link between two concepts indicates that the domain and codomain are “related” to each other, but not by the concept of generality.

SKOS provides a basic vocabulary of metadata terms, which may be used in order to associate lexical labels with resources. Specifically, SKOS allows consumers to distinguish between the “preferred”, “alternate” and “hidden” lexical labels for a given resource. This functionality could be useful in the development of a search engine, where “hidden” lexical labels may be used in order to correct common spelling errors.

As with both DCMI Metadata Terms and OAI-ORE, our decision to use SKOS is motivated by the fact that it is emerging as a *de facto* standard [39]. Moreover, given its overall minimalism, and clarity of design, the SKOS data model is highly extensible, e.g., the semantic relationships that are defined by the SKOS specification may be specialised in order to accommodate non-standard use cases, such as linking concepts according to the similarities of their instances or the epistemic modalities of their definitions.

## Methods and results

In this section, we give three examples of how Semantic Web techniques and technologies can be used in order to support chemistry research: a controlled vocabulary for quantities, units and symbols in physical chemistry; a controlled vocabulary for the classification and labelling of chemical substances and mixtures; and, a database of chemical identifiers. Moreover, we present a Web-based service that uses these datasets in order to assist with the completion of risk assessment forms.

The aim of these datasets is to identify and relate conceptual entities that are relevant to many sub-domains of chemistry, and would therefore, benefit from standardisation. Such conceptual entities are associated with information types that are: requisites for chemistry; understood generally; and available in forms that are amenable to representation using Semantic Web technologies.

Our methodology for the generation of each dataset is to assess the primary use cases, and relate each use case to one or more preexisting vocabularies, e.g., if a dataset relies on the assertion of bibliographic metadata, then we use DCMI Metadata Terms; or, if a dataset requires the aggregation of resources, then we use OAI-ORE. In the event that a suitable vocabulary does not exist, we mint our own.

### IUPAC green book

A nomenclature is a system for the assignment of names to things. By agreeing to use the same nomenclature, individuals within a network agree to assign the same names to the same things, and hence, that if two things have the same name, then they are the same thing. For example, a chemical nomenclature is a system for the assignment of names to chemical structures. Typically, chemical nomenclatures are encapsulated by deterministic algorithms that specify mappings from the set of chemical structures to the set of names. Said mappings need not be one-to-one. In fact, many chemical nomenclatures specify an additional algorithm that computes the canonical representation of a chemical structure before it is assigned a name, resulting in a many-to-one mapping.

The International Union of Pure and Applied Chemistry (IUPAC) develops and maintains one of the most widely used chemical (and chemistry-related) nomenclatures—IUPAC nomenclature—as a series of publications, which are commonly referred to as the “coloured books”, where each book is aimed at a different aspect of chemistry research.

The first IUPAC manual of symbols and technology for physiochemical quantities and units (or “Green Book”) was published in 1969, with the goal of “securing clarity and precision, and wider agreement in the use of symbols by chemists in different countries” [40]. In 2007, following an extensive review process, the third and most recent edition of the Green Book was published.

The goal of this work is to construct a controlled vocabulary of terms drawn from the subject index of the Green Book. If such a controlled vocabulary were available, then researchers would be able to characterise their publications by associating them with discipline-specific terms, whose unambiguous definitions would facilitate the discovery and reuse of said publications by other researchers.

Currently, publications are characterised using terms that are either arbitrarily selected by authors/editors or (semi-)automatically extracted from the content of the publication by software systems [41]. While it has been demonstrated [42,43] that these approaches yield sets of terms that are fit for purpose, it is debatable whether or not the results may be labelled as “controlled vocabularies”, e.g., it has been shown [44] that these approaches are highly susceptible to the effects of user-bias. In contrast, our approach, where terms are drawn from a community-approved, expertly-composed text, yields a true controlled vocabulary.

To typeset the third edition of the Green Book, the authors used the LATE X document mark-up language. From our perspective, this was a fortuitous choice. As the text and typesetting instructions are easily distinguished, the content of a LATE X document is highly amenable to text analysis.

An excerpt of the subject index of the third edition of the Green Book and the corresponding LATE X source is given above. Each term in the subject index is accompanied by zero or more references, where each reference is plain, bold (defining) or underlined (to a numerical entry).

To extract the content of the subject index, we use a combination of two software applications: a lexical analyser (or “lexer”) and a parser. The former converts the input into a sequence of tokens, where each token corresponds to a string of one or more characters in the source that are meaningful when interpreted as a group. The latter converts the sequence of tokens into a data structure that provides a structural representation of the input.

To enrich the content of the subject index: we transform the structural representation into spreadsheets; derive new data; and, generate an RDF graph. First, a spreadsheet is constructed for each of the three entity types: terms, pages and references. Next, using the spreadsheets, we count the number of references per term and page; generate frequency distributions and histograms; and, calculate descriptive statistics. Finally, using a combination of Dublin Core and SKOS, we represent the data as an RDF graph.

**Figure 1:**
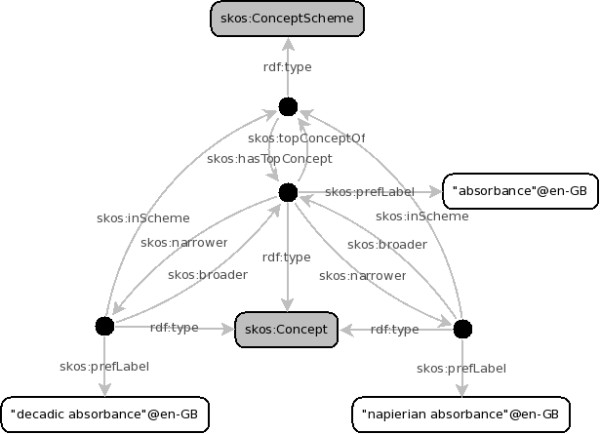
**Depiction of RDF graph that describes three terms from subject index of third edition of IUPAC Green Book.** To construct the graph, we use the SKOS controlled vocabulary, which provides metadata terms for the description of concepts and concept schemes, and the assertion of hierarchical, inter-concept relationships.

A depiction of a region of the RDF graph is given in Figure 1. Each term in the subject index is described by an instance of the skos:Concept class, whose URI is of the form: 

http://id.iupac.org/publications/iupac-books/161/subjects/<Label>

where “Label” is substituted for the URI-encoded version of the lexical label for the term. Lexical labels are also (explicitly) associated with each term using the skos:prefLabel predicate.

The subject index has a tree-like structure, where the “depth” of nodes in the tree corresponds to the “coverage” of terms in the subject index, i.e., that “deeper” nodes correspond to “narrower” terms. To encode the tree-like structure of the subject index, we link terms using the skos:broader and skos:narrower predicates.

To describe the “relatedness” of terms in the subject index, we first index the terms according to their page references and then calculate the set of pairwise cosine similarities. The codomain of the cosine similarity function is a real number whose value is between zero and one inclusive. Pairs of terms with a cosine similarity of exactly one are linked using the skos:related predicate.

In total, we extracted 2490 terms, with 4101 references to 155 of 250 pages in the publication. Despite the fact that it only references only 62% of the pages of the publication, we found that the subject index still has excellent page coverage. Every unreferenced page can be accounted for as being front- or back matter (6%), part of an index (31%) or “intentionally left blank” (less than 1%). During the enrichment phase, we asserted 14154 “relationships” between pairs of terms. Finally, the complete RDF graph contains 40780 triples.

**Figure 2:**
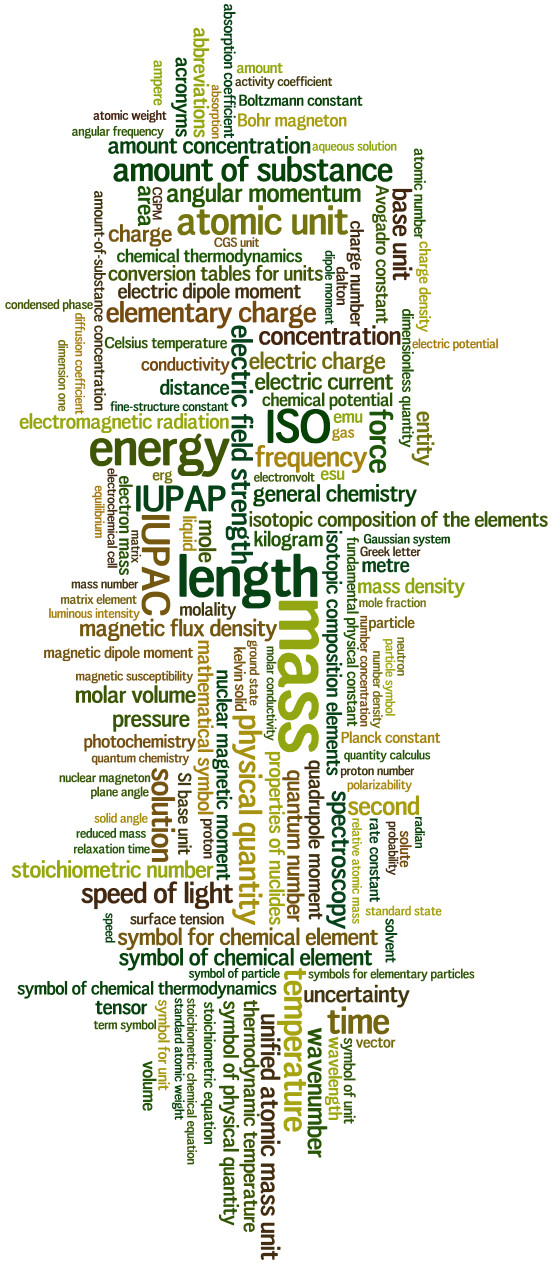
Depiction of weighted word cloud of most frequently referenced terms in subject index of third edition of IUPAC Green Book.

Interestingly, the data can also be used in order to summarise the subject index. A weighted list of the most frequently referenced terms in the subject index is given in Table 1. An alternative—and more aesthetically pleasing—depiction of the same weighted list is given in Figure 2.

**Table 1:** Terms from subject index of third edition IUPAC Green Book with 10 or more references (terms with the same frequency are given in alphabetical order)

**Term**	**Frequency**	**Term (cont.)**	**Frequency**
Mass	29	Solution	12
Length	22	Electric field strength	11
Energy	20	Elementary charge	11
ISO	18	Frequency	11
IUPAC	15	Speed of light	11
Atomic unit	15	Angular momentum	10
IUPAP	14	Base unit	10
Time	14	Concentration	10
Amount of substance	13	Second	10
Temperature	13	Spectroscopy	10
Force	12	Unified atomic mass unit	10
Physical quantity	12	Wavenumber	10

### GHS

The Globally Harmonised System of Classification and Labelling of Chemicals (GHS) is an internationally agreed-upon system for the classification and labelling of chemical substances and mixtures, which was created by the United Nations (UN) in 2005. As its name suggests, the GHS is intended to supersede and harmonise the various systems for classification and labelling that are currently in use, with the goal of providing a consistent set of criteria for hazard and risk assessment that may be reused on a global scale. The manuscript for the GHS, which is published by the UN, is commonly referred to as the “Purple Book” [45].

Following the publication of the GHS, the European Union (EU) proposed the Regulation on Classification, Labelling and Packaging of Substances and Mixtures—more commonly referred to as the “CLP Regulation” [46]. The CLP Regulation was published in the official journal of the EU on 31 December 2008, and entered into legal effect in all EU member states on 20 January 2009. In accordance with EU procedure, the provisions of the CLP Regulation will be gradually phased into law over a period of years, until 1 June 2015, when it will be fully in force.

The CLP Regulation comprises a set of annexes, which are aggregated and disseminated as a single, very large PDF document [47]. The goal of this work is twofold: to use Annexes I, II, III, IV and V—definitions of classification and labelling entities, including: hazard and precautionary statements, pictograms and signal words—in order to construct a controlled vocabulary; and to use Annex VI—a list of hazardous substances and mixtures for which harmonised classification and labelling have been established—in order to construct a knowledge base as an RDF graph.

The primary purpose of this work is to facilitate data integration, whereby organisations that wish to implement the GHS may harmonise their data by relating it to the terms in our controlled vocabulary. However, the work also provides other tangible benefits, e.g., as the data is provided in a machine-processable, language-agnostic format, the development of new, complementary representations and novel software systems is enabled.

Other researches have indicated areas where these capabilities may be beneficial. In their study, Ohkura, et al., describe [48] the need for an alternative representation of the data that is accessible to those with visual impairments. If our controlled vocabulary were used, then it would be trivial to implement a software system that uses speech synthesis to provide an audible version of the GHS. In a separate study, Ta, et al., highlight [49] the high cost of providing localised translations as a key lesson learned from the implementation of the GHS in Japan. If our controlled vocabulary were used, then it would be trivial to associate any number of alternative translations with any term.

**Figure 3:**
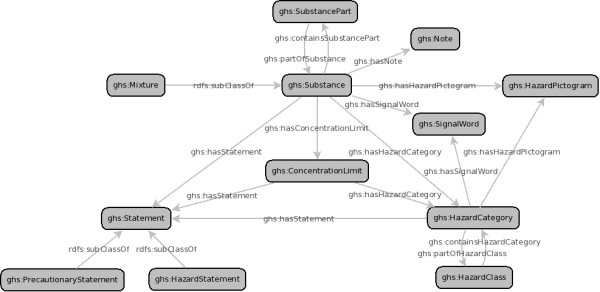
Depiction of RDF schema for core GHS entities and their inter-relationships.

The controlled vocabulary was constructed manually, by reading through the content of Annexes I-V and minting new metadata terms as and when they are were needed. The following URI format was used: 

http://id.unece.org/ghs/<Classglt;/<Label<>

where “Class” and “Label” are substituted for the class name and URI-encoded lexical label for the term. The extraction and enrichment of the content of Annex VI was performed automatically, by processing the PDF document using a text recognition system that was configured to generate data using the controlled vocabulary. A depiction of the entity-relationship model for the core of the controlled vocabulary is given Figure 3.

**Figure 4:**
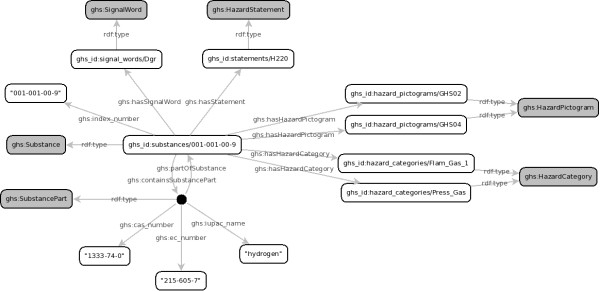
Depiction of RDF graph that describes the chemical substance “hydrogen”.

A key feature is that substances are modelled as aggregations of one or more constituent “parts”. The three main benefits of this approach are as follows: First, metadata can be associated with either the whole or a specific part, e.g., chemical identifiers. Second, using reification, metadata can be associated with the relationship between a whole and a specific part, e.g., volume concentration limits. Finally, by simply counting the number of parts, it is possible to distinguish between substances (of exactly one part) and mixtures (of more than one part). A depiction of the portion of the RDF graph that describes the substance “hydrogen” is given in Figure 4.

Another key feature of our model is that multiple chemical identifiers are used in order to index each chemical substance, including: index number, EC number, CAS registry number and IUPAC name. The main benefit of this approach is that it sharply increases the potential for data integration, where two datasets are joined using a common identifier as the pivot point.

In total, we extracted classification and labelling data for 4136 substances (of which 139 were mixtures) from Annex VI of the CLP Regulation. Finally, the complete RDF graph contains 109969 triples.

### RSC ChemSpider

ChemSpider is an online chemical database [21] that was launched in March 2007. In May 2009, the Royal Society of Chemistry (RSC) acquired ChemSpider. At time of writing, the ChemSpider database contains descriptors of over 26 million unique compounds, which were extracted from over 400 third-party data sources. The ChemSpider database is structure-centric. Every record (a chemical structure) is allocated a locally unique identifier; referred to as a ChemSpider Identifier (CSID).

The core competencies of ChemSpider are: data integration, chemical identifier resolution, and chemical structure search. By associating every unit of information with a CSID, ChemSpider has the capability to extract, enrich and aggregate data from multiple sources. Moreover, ChemSpider has the capability to convert between and resolve many popular chemical identifier formats. Finally, ChemSpider has the capability to locate compounds that match a specified chemical structure or substructure.

**Figure 5:**
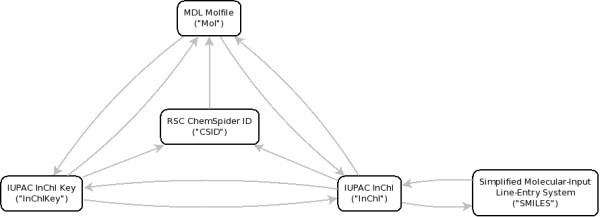
**Depiction of directed graph of RSC ChemSpider “InChI” Web services.** Nodes denote chemical identifier formats. Edges denote the availability of a Web service that provides an injective and non-surjective mapping for chemical identifiers from the source to the target format.

To expose a subset of its capabilities to end users, ChemSpider provides suites of Web services, where each suite of is tailored to a particular use case. For example, the “InChI” suite provides Web services for chemical identifier conversion and resolution [50]. A directed graph, where nodes denote chemical identifier formats and edges denote the availability of a Web service that performs a conversion, is depicted in Figure 5.

Although Web services are provided, the task of incorporating data from ChemSpider into a third party software system is non-trivial. This is because the data has structure but not semantics. Hence, the goal of this work is to construct a RDF graph that describes the content of the ChemSpider database.

In collaboration with the ChemSpider software development team, a model to describe the database was implemented. To describe the chemistry-specific aspects of the data, the ChemAxiom chemical ontology [19] was selected. Use of ChemAxiom affords three key advantages. First, ChemAxiom incorporates the theory of mereology (part-whole relations) and can be used in order to describe (and distinguish between) compounds that consist of more than one moiety. Second, ChemAxiom distinguishes between classes of chemical substances and individual molecular entities. Finally, the design of ChemAxiom is extensible, allowing new aspects of the data to be modelled in the future, e.g., the inclusion of manufacturer- and supplier-specific chemical identifiers.

Records in the ChemSpider database are presented as human-readable Web pages, which are linked to zero or more heterogeneous information resources, including: two- and three-dimensional depictions of the associated chemical structure, chemical identifiers and descriptors, spectra, patents and other scholarly works. To aggregate the information resources into a single, cohesive unit, OAI-ORE was selected.

**Figure 6:**
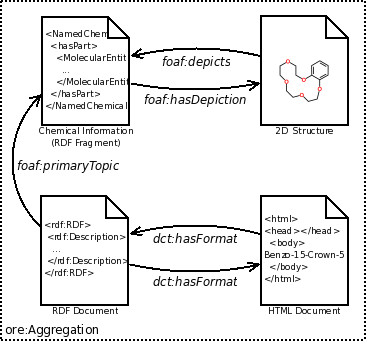
Depiction of OAI-ORE aggregation of information resources associated with an exemplar RSC ChemSpider record.

The main advantage of this approach is that aggregation (as a whole) and its constituent parts can be uniquely identified. Hence, by dereferencing the identifier for the aggregation, users are able to discover all of the associated information resources. A depiction of an OAI-ORE aggregation of the information resources that are associated with an exemplar database record is given in Figure 6. The new, machine-processable, RDF interface to the ChemSpider database was made public in May 2011. Since the announcement [51], the dataset has grown substantially, and now includes synchronised (live) descriptions of every record in the ChemSpider database. At time of writing, this amounts to an RDF graph of over 1.158×10^9^ triples. Finally, an RDF description of the dataset is available at http://www.chemspider.com/void.rdf.

### COSHH assessment form generator service

The Control of Substances Hazardous to Health (COSHH) Regulations 2002 are statutory instruments that govern the use of hazardous substances in the workplace in the UK [52]. COSHH mandates that employers must provide information, instruction and training to any employees who could be exposed to hazardous substances.

A core aspect of COSHH is the requirement for conducting risk assessments. It is recommended that a risk assessment be conducted for each substance that is used in the workplace.

To conduct a risk assessment for a given substance, it is necessary to locate its classification, labelling and packaging information [53]. In the UK, the Chemicals (Hazard Information and Packaging for Supply) (CHIP) Regulations 2009 require that suppliers provide this information in the form of a safety data sheet, which, typically, is included in the packaging, or available via the supplier’s Web site. However, many issues arise when this is not the case, and employees are required to manually locate and/or integrate the necessary information.

Clearly, many of these issues can be addressed with the application of computers. A potential solution could be to implement a software system that assists with the completion of COSHH assessment forms. In principle, to generate a COSHH assessment form, the system would need to cross-reference a set of substances with one or more datasets and then use the results to interpolate a template.

**Figure 7:**
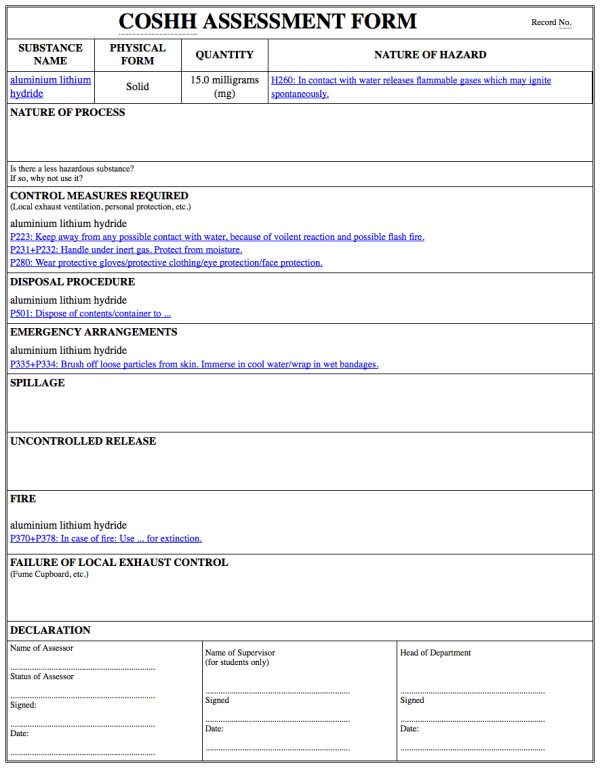
Screen shot of COSHH assessment form generated from GHS description of the chemical substance: “aluminium lithium hydride”.

Accordingly, we have implemented a proof-of-concept of the aforementioned service, where users supply a set of substance-phase-quantity triples. Each triple denotes one substance that will be used as part of the procedure, along with the phase of matter and the amount that will be used (in natural units). The system resolves the chemical identifier for each substance and—when successful—gathers any associated classification and labelling information. Once all the chemical identifiers have been resolved, a template is interpolated, and the result (a partially completed COSHH form) is returned to the user. An exemplar COSHH assessment form, generated by the service for the substance “aluminium lithium hydride”, is given in Figure 7.

Currently, users specify a set of substance-phase-quantity triples, where each substance is denoted by a chemical identifier, which is resolved using RSC ChemSpider, with the result being cross-referenced using the GHS dataset.

In the future, we plan to implement an enhanced version of the service, where the input is a description of a procedure from which the set of the substance-phase-quantity triples is automatically extracted and enriched.

## Legal implications

Following the deployment of the COSHH assessment form generator service, issues were raised about the legal implications of the deployment and the utilisation of an automated system pertaining to health and safety. The issues can be summarised as follows: **Validity** To perform a risk assessment, users of the service must provide a formal description of the procedure that will be preformed (in this case, a set of substance-phase-quantity triples). Given this description, the set of classification and labelling entities can be enumerated, and the form can be generated. However, if we assume that the initial description and the mechanism for generating the form are both valid, then is it correct to infer that the result (the completed form) is also valid? **Accountability** Regardless of the validity of the description of the procedure, who is legally accountable in the event that the information that is asserted by the completed form is incorrect: the third-party, who provided the information; the organisation, who sanctioned the use of the third-party service; or the individual, who accepted the validity of the information? **Value Proposition** Is the net utility that is obtained by the individual, when he/she manually performs a risk assessment, greater than the net utility that is obtained by the organisation, when it delegates the performance of risk assessments to a third-party service provider?

### Validity

The issue of “validity” is deeply important, e.g., within the context of a laboratory environment, the acceptance of, and subsequent reliance on, an “invalid” risk assessment could have negative consequences, including the endangerment of human life. Clearly, “validity” is not the same as “correctness”, e.g., a “valid” risk assessment form is either “correct” or “incorrect”. However, is “invalidity” the same as “incorrectness”?

To provide an answer, we consider the semantics of the term “valid” and its inverse “invalid”. Accordingly, the concept of the “validity” of an artefact (such as a risk assessment form) is defined as follows: An artefact is “valid” if and only if both its constituents and its generator (the mechanism by which said artefact was generated) are “valid”, otherwise, it is “invalid”.

Given this definition, it is clear that, from the point of view of an individual who is employed by an organisation, the “validity” of an artefact must be taken on faith, based on the assumptions that (a) that they are providing “valid” inputs; and (b) their employer has sanctioned the use of a “valid” generator. Similarly, from the point of view of an organisation, the “validity” of an artefact must also be taken on faith, with the assumptions that (c) their employees are providing “valid” inputs; and (d) that the generator is “valid”.

Notice that there are symmetries between assumptions (a) and (c), and assumptions (b) and (d). The symmetry between assumptions (a) and (c) encodes an expectation of the organisation about the future activities of the individual. Similarly, the symmetry between assumptions (b) and (d) encodes an expectation of the individual about the past activities of the organisation.

### Accountability

In the event that any party (the individual, organisation or service provider) has reason to believe that any of the offerings of any of the other parties are “invalid”, then these assumptions are manifest as statements of accountability, responsibility, and ultimately, legal blame. These statements are summarised as follows: 

•An individual is accountable for providing an “invalid” constituent.

•An organisation is accountable for sanctioning the use of an “invalid” generator.

•A service is accountable for providing an “invalid” generator.

Clearly, the truth (or falsity) of these statements could be determined if all of the parties agreed to assert the provenance of their offerings. However, it is important that we consider both the positive and negative effects of the resulting sharp increase in the level of transparency. Essentially, within the context of a provenance-aware software system, if an event occurs, and the system can identify its effects, then the system can also identify its causes (or said differently, within the context of a provenance-aware software system, there is always someone to blame).

### Value proposition

To understand the third issue, a cost-benefit analysis for the deployment and use of a service was conducted from the perspective of the three parties: the individual, the organisation and the service provider.

**Figure 8:**
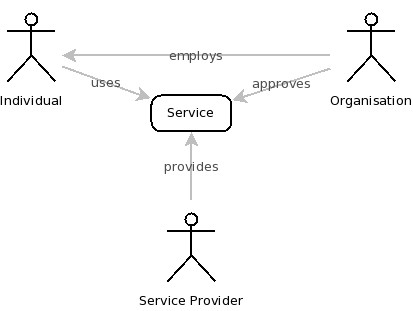
Depiction of the inter-relationships between agents in a service provision scenario.

In Figure 8, we present a depiction of the relationships between the three considered parties. The relationships are summarised as follows:

•The service provider “provides” the service.

•The organisation “approves” (sanctions the use of) the service.

•The organisation “employs” the individual.

•The individual “uses” the service.

From the perspective of an individual (who is employed by an organisation), the benefits of using an automated artefact generation service are that working time will be used more efficiently, and that both the format and information content of artefacts are standardised. In contrast, from the perspective of an individual, the drawbacks of using an automated artefact generation service are an increase in the perceived level of accountability and personal liability.

From the perspective of an organisation (that employs individuals), the benefits of deploying an automated artefact generation service mirror those of the individual. However, from this perspective, the drawbacks of deployment are numerous and varied, e.g., notwithstanding the immediate costs of service deployment and maintenance, and employee training, the organisation also incurs a continuous cost in order to mitigate the risk of employees generating and/or using “invalid” artefacts. Interestingly, as it is possible for the deployment to be managed by a third-party that lies outside of the organisation’s boundary, another drawback of deployment is the potential risk of information leakage.

Finally, from the perspective of the service provider, the benefits of an organisation’s decision to deploy their automated artefact generation service are obvious. First, there is the immediate incentive of financial remuneration for the service provider, e.g., a usage fee. Second, the service provider benefits from brand association and/or co-promotion. However, from this perspective, the drawbacks of the deployment of such a service are also obvious. First, there is the immediate and unavoidable cost of the software development process, and second, there is the risk of the service generating “invalid” artefacts.

The cost-benefit analysis is summarised in Table 2. Given our analysis, we draw the following conclusions: 

•From the perspective of the individual, the costs significantly outweigh the benefits, due to the perception of increased personal liability and legal accountability.

•From the perspective of the organisation, the benefits are balanced by the costs, i.e., while the deployment of the service may improve efficiency and productivity, there are also significant risks associated with the use of automation.

•From the perspective of the service provider, the benefits of financial and marketing opportunities clearly outweigh the costs of development and maintenance.

**Table 2:** Cost-benefit analysis for the deployment and utilisation of an automated artefact generation service, e.g., a service that assists with the completion of risk assessment forms

	**Individual**	**Organisation**	**Service provider**
**Cost(s)**	Increased accountability;Risk of generating (and/or using) an “invalid” artefact;No opportunity to learn (and/or practice) manual artefact generation procedure.	Cost of deployment and maintenance;Cost of employee training;Risk of employees generating (and/or using) “invalid” artefacts;Risk of employees not learning (and/or practicing) manual artefact generation procedure;Risk of employees relying on automated services;Risk of information leakage.	Cost of development and testing;Risk of providing an “invalid” service.
**Benefit(s)**	Increased efficiency and productivity;Quality assurance.	Increased efficiency and productivity;Quality assurance.	Financial incentives (remuneration);Opportunities for marketing, branding and co-promotion.

## Discussion

The development of the IUPAC Green Book dataset has yielded a software tool-chain that can be repurposed for any subject index that is encoded using LATE X document mark-up language. For future work, we intend to apply our approach to the subject indices of the other IUPAC “coloured books”. The resulting controlled vocabularies are useful for data integration and disambiguation, e.g., terms could be used as keywords for scholarly works, enabling “similar” and/or “relevant” scholarly works to be identified. However, as definitions for terms are not provided (the dataset is limited to lexical labels and descriptions of references to the source text), the dataset is not suggestive of other applications.

The development of the GHS dataset has demonstrated the utility that can be obtained when the information content of a legal text is represented using a machine-processable format, where the information content is divided into two categories: definitions and instances, where the latter is represented in terms of the former. In the case of the GHS, or, more specifically, the CLP Regulation, the majority of the text contains definitions. Consequentially, the relatively small number of instances that are provided is not sufficient for use as the primary data source of a software system, such as a COSHH assessment form generator service. While we acknowledge that it would be impossible for any (finite) text to describe (the uncountably infinite set of) every chemical substance, it would be useful if, in the future, the underlying GHS controlled vocabulary could be used in order to describe the product catalogue of a chemical supplier, manufacturer and/or transporter.

More generally, a drawback of our approach is that, currently, the URIs for metadata terms in both the IUPAC Green Book and GHS datasets are non-resolvable. As both datasets are normative, and representative of established, trusted brands, it was decided early on in the project that, rather than minting our own URIs, we should instead assume that the originators will be the eventual publishers, and hence, that the URI schemes for metadata terms in our datasets should be compatible with those that are already in use for human-readable information resources. Given this design decision, it is planned that the datasets be donated to their originators for immediate redistribution (under the umbrella of the originator’s own brand). In the interim, to facilitate the inspection of the IUPAC Green Book and GHS datasets by interested parties, a publicly accessible RDF triple-store has been deployed at http://miranda.soton.ac.uk.

The development of the RDF representation of the content of the RSC ChemSpider database has contributed a significant information resource to the chemical Semantic Web. By leveraging the RDF data, users are able to integrate sources of chemical information by resolving the chemical identifiers to records in the ChemSpider database. Currently, the dataset has two limitations: coverage and availability. First, the descriptions are limited to the chemical identifiers and structure depictions that are associated with each record, representing less than 5% of the available information content. Second, the service does not offer a site-wide daily snapshot or long-term archive. Since we were working in collaboration with the ChemSpider development team, these constraints were outside of our control. However, it is intended that future collaborations address the remaining 95% of the available information content.

Finally, as we have seen, the main issue that was encountered during the development of both the datasets and application was the difficulty of communicating to domain experts the distinction between human judgement and the mechanical application of *modus ponens*. To protect ourselves from any negative effects that may result from a misunderstanding of this distinction, emphasis was placed on the development of a legal framework to support the development of data-driven software systems. However, even with said legal framework in place, it was still difficult to convince some domain experts to trust the data. For future versions, to engineer trust in both the data and its usage by the system, we intend to provide copious amounts of provenance information.

## Conclusions

In the introduction, we set out the importance for the chemistry community of advanced data integration and illustrate the wide acceptance that semantics are necessary to preserve the value of data. Although concerns have been expressed that the lack of robust, usable tools has inhibited the adoption of methodologies based on semantics, recent advances have mitigated those issues.

We have introduced the Semantic Web concepts, technologies, and methodologies that can be used to support chemistry research, and have demonstrated the application of those techniques in three areas very relevant to modern chemistry research, generating three new datasets that we offer as exemplars of an extensible portfolio of advanced data integration facilities: 

•A controlled vocabulary of terms drawn from the subject index of the IUPAC Green Book.

•A controlled vocabulary and knowledge base for the Globally Harmonised System of Classification and Labelling of Chemicals (GHS).

•An RDF representation of the content of the RSC ChemSpider database.

We have implemented a real-world application to demonstrate the value of these datasets, by providing a Web-based service to assist with the completion of risk assessment forms to comply with the Control of Substances Hazardous to Health (COSHH) Regulations 2002, and have discussed the legal implications and value-proposition for the use of such a service. We have thereby established the importance of Semantic Web techniques and technologies for meeting Wild’s fourth “grand challenge”.

## Abbreviations

CAS: Chemical abstracts service; CHESS: Chemical Entity Semantic Specification; CHIP: Chemicals (Hazard Information and Packaging for Supply); COSHH: Control of Substances Hazardous to Health; CSID: ChemSpider Identifier; DCMI: Dublin Core Metadata Initiative; EC: European commission; EPSRC: Engineering and physical sciences research council; EU: European Union; GHS: Globally Harmonised System of Classification and Labelling of Chemicals; IUPAC: International Union of Pure and Applied Chemistry; OAI-ORE: Open Archives Initiative Object Reuse and Exchange; OWL: Web Ontology Language; PDF: Portable document format; RDF: Resource description framework; RDFS: RDF schema; RSC: Royal Society of Chemistry; SKOS: Simple Knowledge Organization System; UK: United Kingdom; UN: United Nations; URI: Uniform Resource Identifier; W3C: World Wide Web Consortium; XML: eXtensible Markup Language.

## Competing interests

The authors declare that they have no competing interests.

## Authors’ contributions

As part of his PhD research, supervised by JGF, MIB initiated the project, constructed the deliverables and encouraged the use cases. All authors contributed to the writing of the paper, and approved the final version. Both authors read and approved the final manuscript.
